# Liver transplantation for homozygous familial hypercholesterolemia: a retrospective analysis from Chinese experience

**DOI:** 10.1186/s13023-024-03443-z

**Published:** 2025-01-15

**Authors:** Hao-Su Zhan, Lin Wei, Wei Qu, Zhi-Gui Zeng, Ying Liu, Yu-Le Tan, Jun Wang, Liang Zhang, En-Hui He, Guang-Peng Zhou, Hai-Ming Zhang, Zhi-Jun Zhu, Li-Ying Sun

**Affiliations:** 1 Department of Critical Liver Diseases, Liver Research Center, Beijing Friendship Hospital, Capital Medical University, Beijing, China; 2 Liver Transplantation Center, National Clinical Research Center for Digestive Diseases, Beijing Friendship Hospital, Capital Medical University, Beijing, China; 3 Clinical Center for Pediatric Liver Transplantation, Capital Medical University, Beijing, China; 4 Department of Anesthesia, Beijing Friendship Hospital, Capital Medical University, Beijing, China; 5 Department of Ultrasound, Beijing Friendship Hospital, Capital Medical University, Beijing, China; 6 Laboratory for Clinical Medicine, Capital Medical University, Beijing, China

**Keywords:** Homozygous familial hypercholesterolemia, Liver transplantation, Children, Long-term prognosis, Metabolic liver disease, Cardiovascular disease

## Abstract

**Background:**

Homozygous familial hypercholesterolaemia (HoFH) increases risk of premature cardiovascular events and cardiac death. In severe cases of HoFH, clinical signs and symptoms cannot be controlled well by non-surgical treatments, liver transplantation (LT) currently represents the viable option.

**Method:**

To assess the clinical efficacy, prognosis, and optimal timing of LT for HoFH, a retrospective analysis was conducted on the preoperative, surgical conditions, and postoperative follow-up of children who received an LT for HoFH at the Beijing Friendship Hospital over the period from December 2014 to August 2022.

**Results:**

Xanthoma and decreased activity tolerance were the primary clinical manifestations in the 7 HoFH children initially assessed (one child died suddenly prior to surgery due to cardiac arrest). Accompanying these symptoms were increased blood total cholesterol (TC) and low density lipoprotein (LDL) levels, along with severe cardiovascular diseases. HoFH was confirmed in all cases by genetic and biochemical assays. Initial treatments administered to these patients consisted of low-fat diets and lipid-lowering drugs with poor outcomes. Accordingly, all 6 patients received orthotopic liver transplantations (OLT), with the result that significant postoperative reductions were observed in levels of TC and LDL. The median follow-up of these six cases was 37.41 months (range: 19.40–94.10 months). Regular postoperative follow-ups revealed that all survived and showed significant improvements in their clinical symptoms.

**Conclusion:**

So far, LT is the only way to heal HoFH. LT before the appearance of obvious cardiovascular atherosclerotic lesions can significantly improve the quality of life and prognosis of patients. At the same time, the blood cholesterol level of patients should be continuously monitored after LT to further control the progression of vascular complications.

## Background

HoFH is an autosomal dominant metabolic disease. This condition is characterized by polymorphisms in the low-density lipoprotein receptor (LDLR) gene and a resultant elevation in serum LDL levels. In HoFH, defects in the typical LDL receptor pathway lead to reduced clearance of LDL, thereby increasing plasma LDL concentrations along with TC levels, eventually leading to valvular heart disease strokes and even the potential for fatal early atherosclerotic cardiovascular disease (ASCVD) [1]. Typical treatments consist of lipid-lowering drugs and lipoprotein apheresis as a means to lower lipid levels. However, recognition of HoFH frequently remains undetected, and its management with these drugs is often suboptimal [2]. In severe cases of HoFH, where blood cholesterol and LDL cannot be controlled by non-surgical treatments, LT currently represents the only viable option [1, 3]. In this report, we review the clinical and follow-up data of HoFH patients receiving LT at our center. The information included in this review consisted of the patients’ demographic characteristics, clinical manifestations, drug treatment regimens, blood test results, surgical data, postoperative treatment regimens, perioperative complications and interventions as well as long-term postoperative follow-up. In this way, we relate our experience and provide a relatively comprehensive analysis regarding clinical evidence for LT as a means for HoFH treatment as performed in China.

### Patients and methods

A retrospective analysis was conducted on the data from 7 HoFH patients. In this review, the preoperative characteristics, surgical conditions, and postoperative follow-up of 6 children (one child died prior to surgery) receiving LT for HoFH as performed at the Beijing Friendship Hospital of Capital Medical University over the period from December 2014 to August 2022 were assessed. The median follow-up of LT cases was 37.41 months (range: 19.40–94.10 months). The information garnered from this article can serve as an important source of clinical evidence and procedures involving LT for the treatment of HoFH. Our center is particularly suited for conducting this review as nearly 1500 LTs, including over 800 pediatric LTs and almost 700 living donor LTs, have been performed at our center [4, 5]. Our study protocol conforms to the ethical guidelines of the 1975 Declaration of Helsinki and was approved by the local ethics committee. Informed consent was obtained from the parents of all patients included in this study.

## Results

### Preoperative patient diagnosis & treatment

A summary of preoperative patient characteristics and surgical procedures employed is contained in Table 1. Six of the 7 patients were male, and case 3 was the younger sister of case 2. The parents of case 1 were cousins, and the parents of the other patients were non-consanguineous. The primary clinical manifestations observed in these patients were a progressive increase in palpable yellow masses within the buttocks and joints and a decreased activity tolerance, accompanied by increased levels of plasma cholesterol and low-density lipoprotein. The diagnosis of HoFH was confirmed from results of genetic and biochemical assay tests. In specific, the genetic test results revealed a compound heterozygous LDLR mutation (*exon6; c.920A > G*) in case 2 and case 3, a compound heterozygous LDLR mutation (*①exon4; c.G665T; ②exon14; c.C2054T*) in case 4, a homozygous LDLR mutation (*exon7; c.G952T > C*) in case 5 and a composite heterozygous LDLR mutation (*①exon5; c.727T > A; ②exon9; c.1187-10G > A*) in case 6. Case 1 completed genetic testing confirmed HoFH without more details. Patients’ mean ± SD preoperative TC level of 16.94 ± 5.73 mmol/L and a blood LDL level of 12.26 ± 3.77 mmol/L. Preoperative treatments consisting of a low-fat diet and lipid-lowering drugs, including rosuvastatin, ezetimibe, probucco and Xuezhikang (doses unknown for some of these drugs), resulted in poor efficacy. LTs were performed at 149, 124, 30, 92, 45 and 72 months after onset of symptoms in cases 1–6, respectively, with all donor livers obtained from donations after circulatory death (DCD).

**Table 1:** Preoperative patient characteristics and operative procedures

Case no.	Sex	Age (yr)			FH gene type		Family history	Non-operation therapy	Prognosis	Follow-up time (m)
		Onset	LT	Current						
1	Male	6	12	19	NA		Mother: Hypercholesteremia Parents: consanguineous marriage	Lipitor 10 mg qn	Alive	94.10
2	Male	4	10	13	LDLR: c.920A > G APOB: c.3607A > G	HeFH	Grandparents and Father: Hypercholesteremia Grandmother: CHD Sister: FH	Rosuvastatin 5 mg qn Ezetimibe 5 mg qd	Alive	34.74
3	Female	5	7	9	LDLR: c.920A > G	HeFH	Grandparents and Father: Hypercholesteremia Grandmother: CHD Brother: FH	NA	Alive	25.80
4	Male	2	7.6	9	LDLR: c.2054C > T c.665G > T	cHeFH	Parents: alive Brother: FH, died at 13	In 2015 Ezetimibe 2.5 mg qd Probucco 63.5 mg qd In Jan 2017 Ezetimibe 5 mg qd Probucco 250 mg qd Rosuvastatin 10 mg qn In July 2017 Ezetimibe 10 mg qd Probucco 250 mg qd Rosuvastatin 10 mg qn In 2019 Ezetimibe 10 mg qd Probucco 250mg qd Rosuvastatin 10 mg qn Xuzhikang 0.3 g qd	Alive	24.73
5	Male	1	3.5	5	LDLR: c.G952T > C	HoFH	Parents: alive Parents and sister: normal cholesterol Grandparents: cardiovascular disease	Ezetimibe Probucco Xuzhikang	Alive	22.97
6	Male	1	6	7	LDLR: c.727T > A c.1187-10G > A	cHeFH	Parents: alive Parents and grandpa: Carry disease genes	Probucco 125 mg bid Xuzhikang 0.6g qd	Alive	19.4
7	Male	16	–	–	LDLR: c.1448G > A c.1845 + 1 G > A	cHeFH	NA	Ezetimibe 10 mg qd Lipitor 10 mg qn PCSK9 inhibitors prn	Died	NA

HoFH: Homozygote familial hypercholesterolemia; HeFH: Heterozygote familial hypercholesterolemia; cHeFH: Compound HeFH; CHD: Coronary heart disease; qd: Once a day; qn: Once every night; bid: Twice a day; PCSK 9: Proprotein convertase subtilisin/kexin type 9; prn: Pro Re Nata(once a while); OLT: Orthotopic liver transplantation; NA: Not available

### Preoperative characteristics, imaging, and test results

Case 1 showed multiple coronary artery stenoses and intra-arterial lipid plaque formation. Case 2 had severe stenosis of the coeliac trunk, multiple stenoses of cervical vessels and experienced chest tightness and precardial pain after exercise. He had a history of acute myocardial infarction before surgery, and coronary angiography showed 100% occlusion of the left anterior descending artery. Case 3 developed multiple xanthoma throughout the body without vascular involvement at first. One year later, he showed atherosclerotic plaque formation in the descending aorta but had no clinical symptoms such as angina pectoris. Case 4 demonstrated a thickened carotid intima-media thickness and plaque formation in the right subclavian artery. Case 5 showed an uneven bilateral thickened intima-media thickness within the external iliac and carotid arteries. Case 6 demonstrated a slight thickened intima-media thickness within the initial portions of both carotid arteries and the right subclavian artery, along with a slight stenosis of the descending aorta. Case 7 had multiple xantomas throughout the body. Echocardiography showed severe valvular disease and decreased ejection fraction, left and right coronary artery stenosis, thickened intima-media thickness in the aorta, carotid artery and cerebral artery, and abdominal aorta aneurysm. A summary of these results is presented in Table 2.

**Table 2:** Preoperative patient vascular involvement

Case No.	Major clinical manifestation		Preoperative mTC/mLDL (mmol/L)	Artery involvement				Ultrasonic cardiogram
	Xanthoma (In order of occurrence)	Others		Brain & neck	Coronary	Abdominal	Limbs	
1	Bilateral hand joints, elbow joints and buttocks	NA	20/13.49	+	+	+	NA	Left atrial enlargement aortic valve stenosis
2	Buttocks and both wrists, elbows, knees, ears	Chest tightness and pain after exercise	12.66/8.99	+	+	+	−	Left atrial enlargement
3	Buttocks and both wrists, ankles	No	22.57/16.03	−	−	+	−	Left atrial enlargement
4	Buttocks	Chest tightness and pain after exercise	12.72/8.96	+	−	−	−	Normal
5	Buttocks and bilateral wrists and ankles	Delayed growth	19.63/13.22	+	NA	NA	+	Normal
6	Buttocks and bilateral wrists and ankles	NA	11.63/8.81	+	+	+	NA	Normal
7	Buttocks, face, corneas, wrists, ankles and fingers	Dyspnea after exercise	15.61/14.76	+	+	+	NA	Aortic valve stenosis and insufficiency. The left atrium and left ventricle were enlarged. The left ventricular ejection fraction was decreased. Pulmonary hypertension

NA: Not available; mTC: Max total cholesterol; mLDL: Max low density lipoprotein

### Clinical changes after surgery

**Fig. 1:**
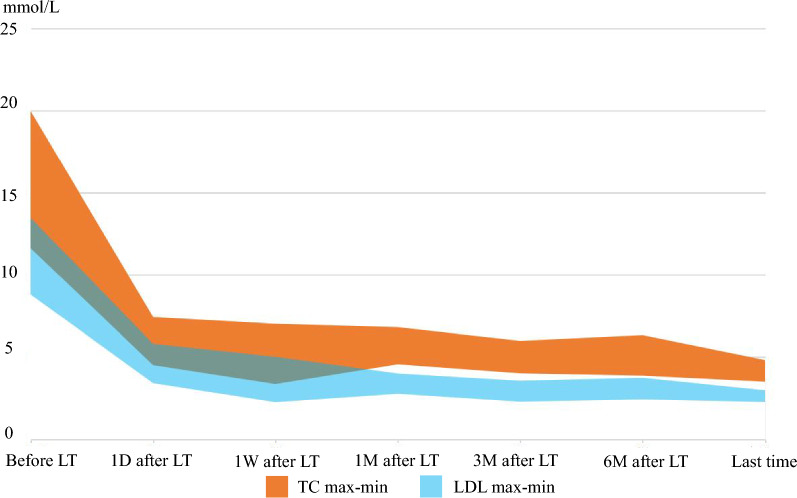
The change range of TC and LDL in children before and after LT

**Fig. 2:**
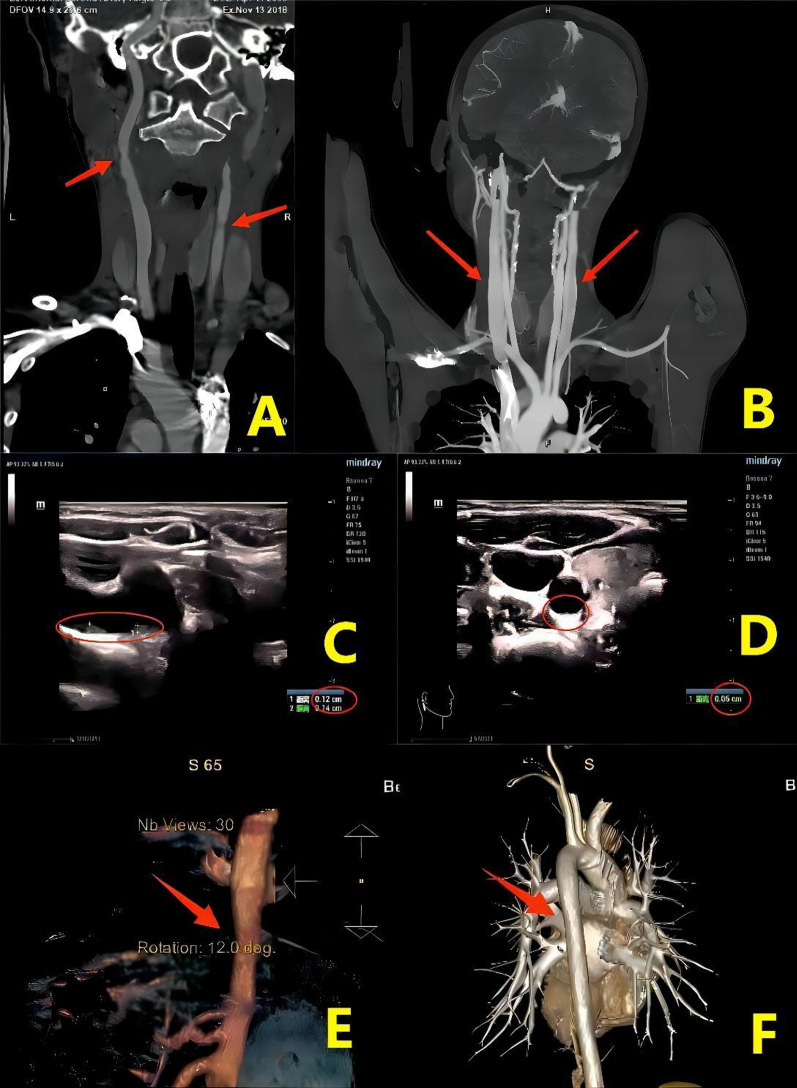
Vascular changes before and after liver transplantation in children. **A** Case 2 carotid artery stenosis (red arrow). **B** The recovered carotid artery in case 2 (red arrow). **C** In case 4, the preoperative carotid intima-media thickness was 0.12 cm (red circle). **D** Carotid artery intima-media thickness of case 4 was about 0.05 cm 3 years after operation (red circle). **E** In case 3, preoperative computed tomography (CT) showed descending aortic plaque (red arrow). **F** In case 3, postoperative CT showed that the descending aorta plaque disappeared (red arrow)

**Fig. 3:**
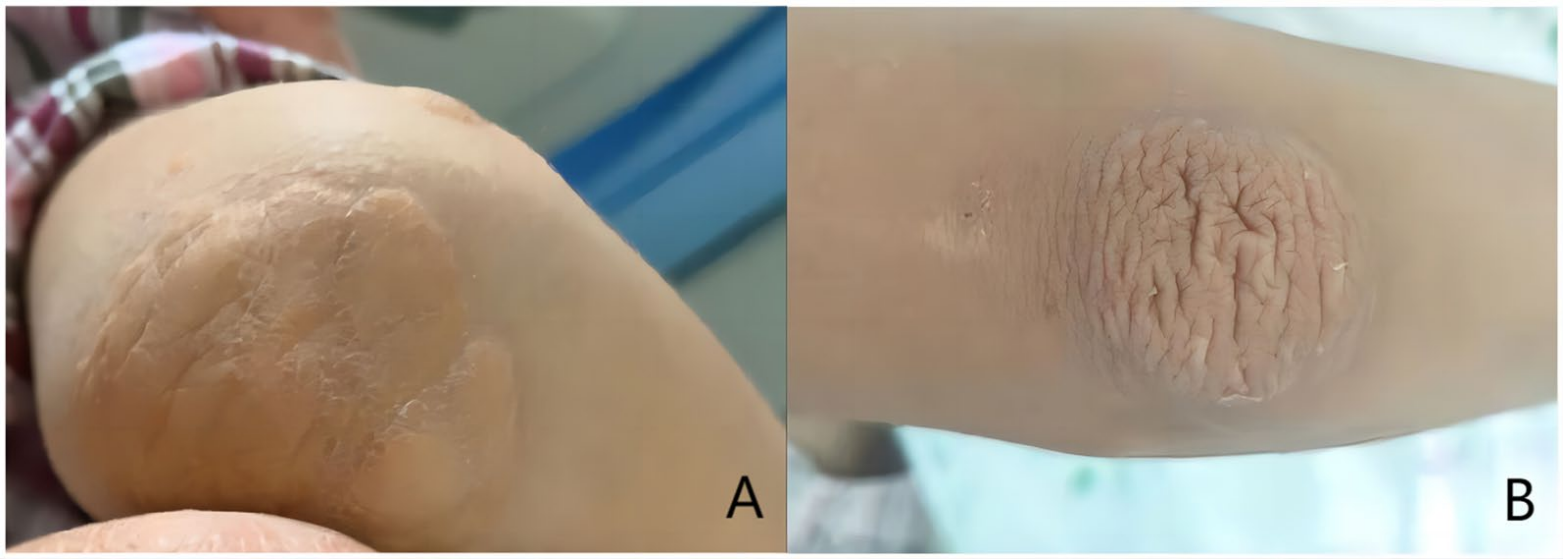
Improvement of cutaneous xantomas in the right elbow in case 2. **A** Preoperative xantomas of the skin in case 2. **B** Case 2 had a regressed xantomas 3 years after transplantation

All 6 children survived the LT surgery and appeared healthy now, and only transient impairment in liver function were observed with a return to normal functioning obtained within a few days to a week. Therefore, LT treatment for HoFH represents a safe and efficacious procedure. On the first day after LT, TC levels were 5.56 ± 1.88 mmol/L and 4.06 ± 1.75 mmol/L for LDL (Fig. 1). There was a gradual recovery of liver function within the 6 patients, blood cholesterol decreased and gradually stabilized within the normal range and normal diets were resumed. Some patients still needed lipid-lowering drugs after operation. Follow ups were conducted at 94.10, 34.74, 25.80, 24.73, 22.97 and 19.4 months for cases 1–6, respectively. Within our patients receiving LT, cutaneous xanthoma was controlled or even dissipated within 3 years after surgery, but improvements in cardiovascular showed a slow recovery. The cardiovascular diseases observed in these 6 children have shown significant improvement as assessed in their postoperative follow-ups so far. The carotid intima-media thickening of cases 2 and 4 were significantly improved, and the descending aortic plaque of case 3completely disappeared (Fig. 2). In case 4, the carotid plaque regressed from 9.7 mm * 1.7 mm to 7.9 mm * 1.1 mm 2 years after surgery. Clinical manifestations such as suffocating, squatting and precardiac discomfort were significantly reduced after LT in these patients, and their cutaneous xanthomas had regressed or even completely dissipated (Fig. 3).

## Discussion

Familial hypercholesterolemia (FH) is an autosomal dominant genetic metabolic disease that can divide into Homozygous FH (HoFH) or heterozygous FH (HeFH). If cholesterol levels cannot be effectively reduced, the child will gradually develop vascular atherosclerosis. In particular, patients with homozygous familial hypercholesterolemia (HoFH) often die from cardiovascular complications around the age of 20.

FH typically results from mutations in four alleles that primarily affect LDLR pathway function, including the low-density lipoprotein receptor, apolipoprotein B (APOB), proprotein convertase subtilisin/kexin type 9 (PCSK9) [6] and LDLR adapter protein 1 (LDLRAP1) genes [7, 8]. Patients demonstrating two or more of these heterozygous genes listed above are clinically classified as compound HeFH (cHeFH), the severity of which was between HoFH and HeFH. While HoFH is considered a rare disease with an incidence of approximately 4–6/million people, HeFH has an incidence of 1/200–500 people. HoFH is more severe than HeFH [9]. It is currently believed that LDL levels are closely related to the genotype and the type of mutation: HeFH < double heterozygote < homozygous APOB or PCSK9 mutation < homozygous LDLRAP1 or LDLR-defective mutations < compound heterozygote LDLR-defective + LDLR-negative mutations < homozygous LDLR-negative mutations. In some patients, although genetic testing results indicated HeFH or cHeFH or double-heterozygous FH, these patients were diagnosed with HoFH in clinical [1, 10]. Severe HeFH patients may have cardiovascular complications and other clinical symptoms with the same severity as that in HoFH. We counted the genotypes of FH patients who underwent liver transplantation in the literature (Table 3).

**Table 3:** Mutant genotypes in FH patients undergoing LT in the existing literature

Literature	Number of Cases	Sex	Age of onset	Types of FH	Mutation gene
[11]	1	M	39 y	cHeFH	LDLR (exon 6 and exon 9)
[12]	1	M	1 y	HoFH	LDLR
[13]	1	F	5 y	HoFH	LDLR (c.1729T > C)
[3]	1	F	15 m	cHeFH	LDLR (c.1068 T > A)
[14]	1	F	3 y	HoFH	NA
[15]	4	F	5 y	HoFH	LDLR (c.996_1011)
		F	3 y	cHeFH	LDLR (c.1103G > A, c.401G > A)
		M	2 y	HoFH	LDLR (c.1061–1G > C)
		M	4 y	HoFH	LDLR (c.1061–1G > C)
[16]	1	M	3 y	HoFH	LDLR (c.1090T > C)
[17]	6	M	2 y	cHeFH	LDLR (c.302A > G, c.1216C > A)
		F	1 y	HoFH	LDLR (c.590G > A)
		M	1 y	HoFH	LDLR (c.2043C > A)
		F	5 y	HoFH	LDLR (c.590G > A)
		F	3 y	HoFH	LDLR (c.2027delG)
		M	1 y	HoFH	LDLR (c.2027delG)
[18]	1	M	4 y	HoFH	LDLR (c.1061–1G > C)
[19]	1	M	41 y	HoFH	LDLR (c.1754T > C)

NA: Not available; M: Male; F: Female; y: Year; m: Month;

As shown in Table 3, not only HoFH, but also cHeFH is the predominant genotype in liver transplant patients in the literature. Their main mutation was LDLR and thesemutations were located in different sites. Meanwhile, new mutation sites were found in the literature. Accordingly, the potential for involvement of other/additional pathogenic genes cannot be ruled out [20–22]. Our results indicate that FH patients caused by LDLR mutations and/or other mutations still require early LT if drug treatment proves ineffective. Even if there are currently no symptoms, close observation about LDL and symptoms are required. If severe cardiovascular involvement occurs, LT should be performed as soon as possible, otherwise combined heart-liver transplantation may be required.

FH patients typically present with symptoms such as xanthomas, corneal arcus, or dyspnea and shortness of breath after exertion during their medical visits. The majority of our patients exhibited reduced exercise tolerance and skin xanthomas. After LT, the xanthomas gradually regressed (Fig. 3).

Early non-surgical treatment options for HoFH generally do not reduce blood cholesterol levels to normal, even with new lipid-lowering drugs [23–25]. All our patients had been treated with lipid-lowering strategies with limited beneficial effects, and worsening of their cardiovascular complications was observed. Therefore, LT was required.

As LDL receptors in the donor liver begin to function immediately, HoFH patients show a very rapid resumption of normal blood cholesterol levels following LT [26, 27], in some cases even on the first day after surgery (Fig. 1). Subsequently, their dermal xanthoxoma corneal arch recedes, activity tolerance and other clinical symptoms are also significantly improved. Results from imaging reveal that the degree of arterial stenosis does not progress or is slowed down, and even varying degrees of regression may be observed. In our cases, following LT, cardiovascular complications were relieved to varying degrees, and some blood vessels resumed normal structures (Fig. 2). In our cases, the cutaneous xanthoma gradually regressed after LT (Fig. 3). Limited studies on long-term survival after treatment of HoFH with LT are available. Results from an Iranian study [28, 29] indicated that survival rates in HoFH patients after LT at 0.5, 1 and 5 years were 92%, 94% and 91%, respectively. In another case on the long-term survival in a HoFH patient whose skin xanoma and atherosclerotic plaques were completely resolved at 9 years after LT and endothelial function was normal [3]. The clinical status of this patient remained stable for 14 years after LT, but LDL levels gradually increased. After the patient resumed normal levels with the addition of appropriate lipid-lowering medications.

In our study, patients with severe and multi-site vascular complications had much worse recovery than those with only mild and few sites of vascular complications. And they had poor performance in terms of atherosclerotic plaque regression and postoperative quality of life, so that their improvement wasn’t significant or even died before LT. Patients with mild and few vascular complications even showed a complete disappearance of atherosclerotic plaque in postoperative imaging. Unfortunately, one child (case 7) died from cardiac arrest due to severe cardiovascular disease prior to LT. Therefore, we believe that closely monitoring LDL levels and the occurrence of atherosclerosis in FH patients is essential. However, when non-surgical treatments fail to achieve optimal results, early listing for LT should be considered to avoid combined heart-liver transplantation or fatal cardiovascular and cerebrovascular events.

In our patients, those with a shorter waiting time from symptom onset to LT had better prognosis and cardiovascular recovery. These patients may still have mildly elevated cholesterol after LT. We recommend that patients continue to monitor the changes in cholesterol after LT to add lipid-lowering drugs in time to prevent disease progression when cholesterol fluctuation occurs.

Currently, all 6 LT patients remain healthy and we continue to monitor their progress to assess the long-term effects.

## Conclusion

So far, LT is the only way to heal HoFH. Performing LF before the onset of significant vascular atherosclerotic lesions can significantly improve the patient's quality of life and prognosis. At the same time, the blood cholesterol level of patients should be continuously monitored after LT to further control the progression of vascular complications.

## Data Availability

The data that support the findings of this study are not openly available due to sensitivity reasons and are available from the corresponding author upon reasonable request. Data are located in controlled access data storage at Beijing Friendship Hospital, Capital Medical University.

## Abbreviations

LT: Liver transplantation
FH: Familial hypercholesterolemia
HoFH: Homozygous familial hypercholesterolemia
LDL: Low density lipoprotein
ASCVD: Atherosclerotic cardiovascular disease
LDLR: Low-density lipoprotein receptor
TC: Total cholesterol
DCD: Donations after circulatory death
HeFH: Heterozygous familial hypercholesterolemia
APOB: Apolipoprotein B
PCSK9: Proprotein convertase subtilisin/kexin type 9
LDLRAP1: Low-density lipoprotein receptor adapter protein 1
cHeFH: Compound heterozygous familial hypercholesterolemia
OLT: Orthotopic liver transplantation
CT: Computed tomography
